# Association between Physical Activity and Teacher-Reported Academic Performance among Fifth-Graders in Shanghai: A Quantile Regression

**DOI:** 10.1371/journal.pone.0115483

**Published:** 2015-03-16

**Authors:** Yunting Zhang, Donglan Zhang, Yanrui Jiang, Wanqi Sun, Yan Wang, Wenjuan Chen, Shenghui Li, Lu Shi, Xiaoming Shen, Jun Zhang, Fan Jiang

**Affiliations:** 1 Shanghai Jiao Tong University School of Public Health, Shanghai, China; 2 Child Health Advocacy Institute, Shanghai Children's Medical Center, Shanghai Jiao Tong University School of Medicine, Shanghai, China; 3 Department of Health Policy and Management, UCLA Fielding School of Public Health, Los Angeles, United States of America; 4 Department of Developmental and Behavioral Pediatrics, Shanghai Children’s Medical Center, Shanghai Jiao Tong University School of Medicine, Shanghai, China; 5 Department of Public Health Sciences, Clemson University, Clemson, United States of America; 6 Ministry of Education-Shanghai Key Laboratory of Children’s Environmental Health, Xinhua Hospital, Shanghai Jiao Tong University School of Medicine, Shanghai, China; University Of São Paulo, BRAZIL

## Abstract

**Introduction:**

A growing body of literature reveals the causal pathways between physical activity and brain function, indicating that increasing physical activity among children could improve rather than undermine their scholastic performance. However, past studies of physical activity and scholastic performance among students often relied on parent-reported grade information, and did not explore whether the association varied among different levels of scholastic performance. Our study among fifth-grade students in Shanghai sought to determine the association between regular physical activity and teacher-reported academic performance scores (APS), with special attention to the differential associational patterns across different strata of scholastic performance.

**Method:**

A total of 2,225 students were chosen through a stratified random sampling, and a complete sample of 1470 observations were used for analysis. We used a quantile regression analysis to explore whether the association between physical activity and teacher-reported APS differs by distribution of APS.

**Results:**

Minimal-intensity physical activity such as walking was positively associated with academic performance scores (β = 0.13, SE = 0.04). The magnitude of the association tends to be larger at the lower end of the APS distribution (β = 0.24, SE = 0.08) than in the higher end of the distribution (β = 0.00, SE = 0.07).

**Conclusion:**

Based upon teacher-reported student academic performance, there is no evidence that spending time on frequent physical activity would undermine student’s APS. Those students who are below the average in their academic performance could be worse off in academic performance if they give up minimal-intensity physical activity. Therefore, cutting physical activity time in schools could hurt the scholastic performance among those students who were already at higher risk for dropping out due to inadequate APS.

## Introduction

The resource allocation between children’s academic learning and their physical activity has been believed by many to be a zero-sum game [1]. However, a lot of evidence has been published to prove that spending time on physical activity would not necessarily undermine children’s academic achievements. A systematic review of free school physical activity concluded that free school physical activity was positively associated with better academic outcomes, suggesting physical activity may enhance scholastic performance [2]. A randomized experimental study conducted among sixth-grade students in the US found that students who performed vigorous activity had significantly higher grades, and moderate physical activity did not affect grades [3].For the younger population, Drollette et al [4] found that preadolescent children’s participation in a single bout of structured physical activities lasting at least 20 minutes is beneficial for various cognitive functions, including aspects of concentration, reading and mathematics achievement, and inhibitory control. As an interpretation for this consistent link between exercise and brain function, it is hypothesized physical exercise could up-regulate neurotrophins and induce neuronal growth [5].Evidence from neuroscience proves the point: increases in cardiovascular fitness results in increased functioning of the brain’s attentional network [6]; exercise training has been shown to increase the size of hippocampus and improves memory [7].Among physically-disabled youth, physical activity may increase baseline neuronal activity, and help improve brain health [8].

Not all studies of this topic have shown a positive association between physical activity and enhanced academic performance. Some studies found non-significant or negative associations between physical activity programs in schools and several aspects of academic achievement (such as math scores and language scores) [9–11]. Moreover, home environment, health status, and school-specific factors may potentially confound the observed positive relationships. For instance, family socioeconomic status simultaneously affects child pre-academic skills, self-regulation, math skills, and health behaviors such as leisure-time physical activities [12–13]. Students with health problems may be less likely to participate in physical activity [14] and their health problems may interfere with academic achievement as well [15]. Additionally, the observed positive association may depend largely on the particular school environment. School engagement, which includes teacher support and teacher-parent communication, were important determinants of student’s academic performance [16], and such school-level factors also influenced frequency of students’ physical activity [17]. Furthermore, it is not clear in the literature whether the positive relationship between physical activity and academic performance varies by intensity of exercise or differs by distribution of academic performance scores. Finally, the fact that many such studies use parent-reported or students’ self-reported information about the student scholastic performance casts doubt upon the reliability of the scholastic performance measure, and thus could compromise the policy implication of the significant associations they identified.

This study is partly driven by China’s recent decline in physical fitness including vital capacity, speed, strength, and endurance from 1985 to 2005 among school-age children [18–19]. Along with this trend, prevalence of overweight and obesity among school children in China’s large cities was increasing rapidly [20]. To address these issues, the Chinese government has taken measures to improve physical education in schools. In 2010, Ministry of Education mandated physical education in schools to improve the physical health of the students. The Ministry regulated the frequency of physical education classes and duration of in-class and after-class physical activities [21]. However, China’s current education system strongly prioritizes student’s academic performance [22]. As physical education is not a primary requirement for China’s college admission competition, it has been given a low priority among China’s school principals [23]. Consequently, physical education classes often gave way to other classes, and exercise time in- and after-school is often filled with extracurricular studying activities [24].

In this study, we use teacher-reported scholastic performance information of students to explore the association between the frequency of physical activity and academic performance among fifth-grade students. We attempt to fill a research gap by exploring whether this association differs by intensity of physical activity and varies across students with different strata of academic performance. Given our more objective measure of students’ academic performance and more in-depth analysis of the data, this study is likely to contribute new insights for those who wonder how much of the child’ time should be given to physical activity. For school principals and school district policy makers, our findings would be helpful in planning school policies on students’ time allocation between physical activity and academics.

## Methods

### Study Population

This was a cross-sectional study with all data collected in 2009. Institutional Review Board approval for this study was obtained from the Shanghai Children’s Medical Center. The study was conducted in public schools in Shanghai, China. The study sample was obtained using stratified random sampling technique. The city of Shanghai was stratified into 18 administrative units including urban districts and suburban regions, 10 administrative units were designated as primary sampling units (PSUs). In each PSU, one public school was selected. We conducted interviews using a structured questionnaire with all fifth-graders in those 10 public schools and with one of their parents. Informed consent was obtained from the interviewees. Fifth-graders were chosen because they were about to take an entrance examination to enter a middle school, and we assumed they would be highly motivated to improve their academic performance. Data on students’ academic performance was obtained separately from their class head teachers. The average class size was 40 students per classroom in those public schools. Ninety-eight percent of the participants completed the questionnaire, resulting in a total sample size of 2,225 respondents.

### Variable Measurement

The International Physical Activity Questionnaire (IPAQ) (Chinese version) was used to measure students’ weekly physical activity (PA) levels [25]. Each student was asked: “Within the last 7 days, about how many days did you participate in vigorous/moderate/minimal (i.e. walking) physical activities that last for at least 10 minutes?” Among those whose answers were greater than “0” days to the above question, students were further asked “On those days when you participated in physical activities, about how many minutes did you spend on vigorous/moderate/minimal (i.e. walking) physical activities each day?” A baseline assessment by the research team showed that measures on the second question—minutes of physical activities per day were of poor quality. 33.62% of the students did not answer the latter question or could not remember the number of minutes of physical activity during the past 7 days. Thus, we only chose the measures of the frequencies of PA (days/week) as primary predictors.

The outcome variable in this analysis was academic performance scores (APS). The questionnaire was developed based on the Oliviero Bruni’s Teacher school achievement form [26].It was composed of six items covering different aspects related to school achievements such as reading ability, reading comprehension, mathematics, executive ability, interest and attention. We assessed components of students’ listening and speaking skills, reading skills, writing skills, mathematical calculation and mathematical logical reasoning evaluated by their head teachers, who had supervised the student for more than one year. Each component was measured by a 5-point rating scale, with higher scores indicating better performances. Reliability of the questionnaire was tested in another paper using a pilot sample, where the overall Cronbach α coefficient for this questionnaire was 0.962, and the Cronbach α for each dimension used in this study ranges from 0.905 to 0.961, indicating good reliability [27]. Scores on each of those components were summed and used as the outcome measure.

Adjustments were made for a series of potentially confounding factors in the regression analysis: student’s demographic characteristics, parents’ socioeconomic status, time spent on other activities and health conditions. Demographic characteristics included the student’s age and gender. Information on parents’ socioeconomic status was obtained from the parents’ questionnaire, which included measures of household income per capita, family structure and parents’ educational attainment. Income per capita in the household was a categorical variable with income less than ¥1500 ($245) per month as the reference group. Another two groups were income ranges from ¥1500- ¥2500 ($245 –$408) and income greater than ¥2500 ($408) per month, with reference to Shanghai Bureau of Statistics on the categories of lower-middle, middle and higher-middle families defined by monthly disposable income per capita in 2009 [28]. Family structure was categorized into nuclear family (the child living with both parents: the reference group), single-parent family (the child living with a single parent) and extended family (the child living with both parents and grandparents). Parents’ educational attainment was measured by the father and the mother’s education levels, categorized into less than high school (the reference group), finished high school and attended college or above.

In addition, there is a trade-off between time spent on PA and on other activities that are related to academic performances. Those activities may include doing homework and participating in extra-curricular activities. We included both variables in this study. The students were asked, “How many hours did you spend on doing homework every day?” The variable was then categorized into four groups with “less than 1 hour” as the reference group, and “1–2”, “2–3” and “>3” hours as the other groups. The students were also queried about whether they attended extra-curricular activities. Those who answered “No” were in the “0” (reference) group, and those with the “Yes” answers were in the “1” group.

Students’ health status may be associated with frequency of PA as well as cognitive outcomes. We thus further controlled for students’ weight status, whether or not the student suffered from allergic rhinitis or asthma. With the exception of students’ weight, all the information was collected from the parent’s questionnaire. Height and weight were measured with the subjects wearing light clothes and no shoes. Height was measured to the nearest 0.1 cm with a stadiometer (RGY-140 stadiometer, Shanghai BaoShan Weighter Factory). Weight was measured to the nearest 0.1 kg with a weighing scale (Type RGT-140-RT Body Weight Balance, WuXi Weigher Factory Co. Ltd.). All assessments were performed twice, and their average was used in the analyses. BMI was calculated as weight (kg)/height squared (m^2^).We defined obesity status using P(95) as the cut-off point based upon the BMI-for-age percentiles for Chinese children and adolescents established by the group of China Obesity Task Force [29].

School-level factors may also confound the association of interest. However, the study did not focus on identifying any specific school-level factor. School-level factors were treated as fixed effects in our analysis.

### Data Analysis

Ordinary least squares (OLS) regressions were performed to examine the cross-sectional association between PA and APS, adjusting the aforementioned confounders including student’s age and gender, family income per capita, parents’ education, time spent on other activities and their health conditions [12–17]. Students’ APS was regressed separately on frequencies of different levels of PA including vigorous-intensity physical activity (VIPA), moderate-intensity physical activity (MoIPA) and minimal-intensity physical activity (MiIPA). School effects were added into the regression to account for omitted-variable bias at the school-level. To determine if a fixed effects model is preferred to a random effects model, we conducted a Hausman-Wu test to compare the school fixed effects with random effects regression. The Hausman-Wu test showed that we should reject the null hypothesis that the random-effects estimates were consistent with the fixed-effects estimates (P<0.001). We then proceeded to use the linear regression with school fixed effects. To explore how PA was associated with the distribution of APS, we applied a quantile regression analysis with school effects included. Quantile regression is an extension to OLS regression. But differs from the classical OLS regression, which is a conditional mean function that describes how the mean of dependent variable changes with respect to a vector of covariates, and also assumes the error has the same distribution for all values of the covariates (i.e. homoscedasticity), quantile regression aims at estimating either the conditional median or other quantiles of the outcome variable, which offers a complete view of the effect of the covariates on the distribution of the dependent variable [30]. One hundred bootstrap replications were performed in the quantile equations to compute the standard errors (SE) of the estimators. Post-estimation tests were performed and results were plotted to check the equivalences of coefficients at various quantiles. Statistical significant tests were performed at α = 0.05 level. All analyses were conducted in STATA/SE 12 (StataCorp LP, College Station, Texas, USA).

## Results

Characteristics of the sample were depicted in Table 1. The mean APS for the study sample was 17.94, whereas the 25^th^ percentile was 15, the median was 18, and the 75^th^ percentile was 20. The average frequency of PA was 2.25 days per week for VIPA, 3.09 days per week for MoIPA, and 5.23 days per week for MiIPA. Demographic information included: the students were 10.33 years old on average; 49.44% (N = 1052) were boys; 19.78% (N = 416) had household income per capita less than ¥1500 per month; 5.42% (N = 115) lived in a single-parent family; 24.80% (N = 524) of their mothers and 19.98% (N = 422) of their fathers had not completed high school; 67.62% (N = 1443) of the students attended extracurricular activities; 11.40% (N = 244) spent more than three hours on homework each day. Health status included: 11.18% (N = 194) were obese, 15.49% (N = 330) had chronic allergic rhinitis and 4.36% (N = 93) had asthma. Except for weight status, all variables contained less than 6% missing values. Weight status was missing for 22.02% of the sample. To test if it was missing completely at random, we created a dummy variable to indicate whether the weight measure was missing, and ran a cross-table check about the correlation between the indicator variable and the other variables. We detected no significant results from the correlation tests. Finally, a complete sample of 1470 observations was used for analysis.

**Table 1:** Descriptive Statistics of the Study Sample (Year = 2009).

Variables	All	Quantiles				
Mean(SD)/Frequency(%)		10^th^ quant.	25^th^ quant.	Median	75^th^ quant.	90^th^ quant.
**N**	2225	674	439	538	347	227
**Academic Performance Scores**	17.94(4.13)	13.15 (2.45)	17.10 (0.83)	19.71 (0.45)	22.19 (1.11)	25 (0.00)
***Primary Regressors of Interest***						
**VIPA**	2.25 (2.16)	2.34 (2.12)	2.07 (2.20)	2.02 (2.08)	2.64 (2.28)	2.28 (2.13)
**MoIPA**	3.09 (2.37)	2.80 (2.42)	3.24 (2.27)	3.07 (2.32)	3.67 (2.38)	2.86 (2.35)
**MiIPA**	5.23 (2.43)	4.87 (2.64)	5.30 (2.43)	5.45 (2.28)	5.47 (2.24)	5.31 (2.27)
***Demographic and Socioeconomic Characteristics***						
**Age**	10.33 (0.61)	10.33(0.89)	10.32 (0.39)	10.34 (0.54)	10.31 (0.37)	10.37 (0.39)
**Gender**						
Boys	1052(49.44%)	357 (55.61%)	216 (51.67%)	231 (44.94%)	159 (47.46%)	89 (40.64%)
Girls	1076(50.56%)	285 (44.39%)	202 (48.33%)	283 (55.06%)	176 (52.54%)	130 (59.36%)
**Household Income Per Capita Per Month**						
<¥1500	416 (19.78%)	155 (24.56%)	83 (19.90%)	98 (19.33%)	43 (12.99%)	37 (17.05%)
¥1500–2500	516 (24.54%)	164 (25.99%)	110 (26.38%)	121 (23.87%)	64 (19.34%)	57 (26.27%)
>¥2500	1171(55.68%)	312 (49.45%)	224 (53.72%)	288 (56.80%)	224 (67.67%)	123 (56.68%)
**Family Structure**						
Single-parent	115 (5.42%)	47 (7.34%)	28 (6.71%)	17 (3.32%)	11 (3.28%)	12 (5.53%)
Nuclear family	1272(59.97%)	386 (60.31%)	241 (57.79%)	307 (59.96%)	212 (63.28%)	126 (58.06%)
Extended family	734 (34.61%)	207 (32.34%)	148 (35.49%)	188 (36.72%)	112 (33.43%)	79 (36.41%)
**Mother’s educational attainment**						
Less than high school	524 (24.80%)	203 (31.92%)	107 (25.72%)	124 (24.36%)	48 (14.37%)	42 (19.27%)
High school	592 (28.02%)	223 (35.06%)	123 (29.57%)	123 (24.17%)	74 (22.16%)	49 (22.48%)
College and above	997 (47.18%)	210 (33.02%)	186 (44.71%)	262 (51.47%)	212 (63.47%)	127 (58.26%)
**Father’s educational attainment**						
Less than high school	422 (19.98%)	166 (26.06%)	95 (22.89%)	96 (18.75%)	37 (11.11%)	28 (13.02%)
High school	645 (30.54%)	225 (35.32%)	131 (31.57%)	158 (30.86%)	75 (22.52%)	56 (26.05%)
College and above	1045(49.48%)	246 (38.62%)	189 (45.54%)	258 (50.39%)	221 (66.37%)	131 (60.93%)
***Time spent on other activities***						
**Attend Extracurricular Activities?**						
Yes	1443(67.62%)	364 (57.23%)	276 (64.64%)	367 (71.26%)	268 (79.29%)	168 (77.06%)
No	691 (32.38%)	272 (42.77%)	151 (35.36%)	148 (28.74%)	70 (20.71%)	50 (22.94%)
**Average Time Spent on Homework Per Day**						
< 1 hour	188 (8.79%)	28 (4.33%)	33 (7.80%)	47 (9.16%)	52 (15.38%)	28 (12.79%)
1–2 hours	947 (44.25%)	211 (32.61%)	191 (45.15%)	258 (50.29%)	163 (48.22%)	124 (56.62%)
2–3 hours	761 (35.56%)	291 (44.98%)	147 (34.75%)	172 (33.53%)	96 (28.40%)	55 (25.11%)
> 3 hours	244 (11.40%)	117 (18.08%)	52 (12.29%)	36 (7.02%)	27 (7.99%)	12 (5.48%)
***Health Status***						
**Weight Status**						
Obese	194 (11.18%)	72 (13.38%)	32 (9.30%)	52 (12.47%)	22 (8.49%)	16 (9.04%)
Not Obese	1541(88.82%)	466 (86.62%)	312 (90.70%)	365 (87.53%)	237 (91.51%)	161 (90.96%)
**Having Chronic Allergic Rhinitis?**						
Yes	330 (15.49%)	95 (14.87%)	64 (15.02%)	69 (13.45%)	71 (21.19%)	31 (14.22%)
No	1801(84.51%)	544 (85.13%)	362 (84.98%)	444 (86.55%)	264 (78.81%)	187 (85.78%)
**Having Asthma?**						
Yes	93 (4.36%)	27 (4.21%)	13 (3.07%)	19 (3.69%)	18 (5.39%)	16 (7.31%)
No	2041(95.64%)	615 (95.79%)	411 (96.93%)	496 (96.31%)	316 (94.61%)	203 (92.69%)

Note: Missing values for Academic performance scores were 60 (percent of missing = 2.70%), for age were 74 (percent of missing = 3.33%), for gender were 97 (percent of missing = 4.36%), for household income were 122 (percent of missing = 5.48%), for family structure were 104 (percent of missing = 4.67%), for mother’s education were 112 (percent of missing = 5.03%), for father’s education were 113 (percent of missing = 5.08%), for attending extracurricular activities were 91 (percent of missing = 4.09%), for average time spent on homework were 85 (percent of missing = 3.82%), for weight status were 490 (percent of missing = 22.02%), for rhinitis were 94 (percent of missing = 4.22%) and for asthma were 91 (4.09%).

Tables 2–4 presented the results of linear regression, linear regression without school effects, and quantile regression with school fixed effects. The estimated standard errors were presented in parentheses. In the OLS regressions without school effects, VIPA was found to be negatively and significantly associated with APS (β = -0.22, SE = 0.05), whereas MoIPA had no significant association (β = 0.02, SE = 0.04). MiIPA had a significant positive association with APS (β = 0.12, SE = 0.04), when adjusted for demographic, family socioeconomic status, health status, and time spent on other activities. But the significant association between VIPA and APS disappeared after controlling for school fixed effects (β = -0.02, SE = 0.05). The other two associations did not change significantly when we included school fixed effects. Besides frequency of physical activities, time spent on homework was negatively associated with students’ academic performance, whereas attending extracurricular activities was positively associated with APS. We were particularly interested in the results found when we investigated the heterogeneous association with different parts of the conditional APS distribution in the quantile regressions. MoIPA and MiIPA played a different role in students’ APS quantiles. We found the magnitude of the estimates decreased monotonically from a lower quantile to a higher quantile of APS. There was a statistically significant and positive association between MoIPA and APS and between MiIPA and APS if the students were in the lower half of the APS distribution (10% and 25%), while this was not the case any more for students who belonged to the top 50% of APS distribution in our sample (50%, 75% and 90%). In particular, the quantile regression coefficient of MoIPA at the 25^th^ percentile of academic performance suggested that every one day increase in moderate physical activity per week was associated with a 0.15 point or 0.6% ((0.15/25)*100 = 0.6) increase in APS, after controlling for all other factors in the model. The quantile regression coefficient of MiIPA showed that at the 10^th^ and 25^th^ percentiles of APS, a one-day increase in walking for at least 10 minutes per week, predicted an increase in the academic scores by 0.24 points (1.0%) and 0.17points (0.7%) respectively, holding all else constant in the model. A post-estimation test suggested that the coefficients of VIPA were not significantly different across different quantiles of the APS (F = 1.42, P = 0.22). However, for MoIPA, the coefficient at the 25^th^ percentile of APS was significantly different from that at the 90^th^ percentile (F = 6.31, P = 0.01). And for MiIPA, the coefficients at the 10^th^ and 25^th^ percentile were different from that at the 90^th^ percentile (F = 7.02, P = 0.01 and F = 4.41, P = 0.04).

**Table 2:** Regression Results of the Relation between Frequency of Vigorous-Intensity Physical Activity (VIPA, days/week) and Distribution of Academic Performance Scores (N = 1470, Year = 2009).

	OLS Regression		Quantile Regression with School Effects^1^				
	Without School Effects	With School Effects	10^th^ quant.	25^th^ quant.	Median	75^th^ quant.	90^th^ quant.
	*β*(SE)	*β*(SE)	*β*(SE)	*β*(SE)	*β*(SE)	*β*(SE)	*β*(SE)
**VIPA**	-0.22*** (0.05)	-0.02 (0.05)	-0.11 (0.10)	-0.08 (0.08)	-0.09 (0.08)	0.07 (0.06)	0.04 (-0.11)
**Age**	0.10 (0.15)	0.07 (0.14)	-0.02 (0.55)	0.13 (0.40)	0.24 (0.39)	0.38 (0.41)	0.53 (-0.02)
***Demographic and Socioeconomic Characteristics***							
**Gender (Reference—Male)**							
Female	0.67*** (0.21)	0.76*** (0.19)	0.61 (0.36)	0.42 (0.32)	0.47 (0.28)	0.76** (0.24)	1.05**(0.61)
**Household Income Per Capita Per Month (Reference—>¥2500)**							
<¥1500	0.09 (0.31)	-0.55 (0.30)	-0.43 (0.51)	-0.39 (0.49)	-0.84* (0.35)	-0.72 (0.37)	-1.30 (-0.43)
¥1500–2500	0.36 (0.27)	0.02 (0.26)	0.17 (0.42)	-0.07 (0.32)	-0.01 (0.30)	-0.08 (0.30)	-0.07 (0.17)
**Family Structure (Reference—Nuclear family)**							
Single-parent	-0.36 (0.47)	-0.54 (0.45)	-0.56 (0.71)	-0.92 (0.65)	-0.84 (0.64)	-0.49 (0.86)	-0.06 (-0.56)
Extended family	0.05 (0.22)	-0.03 (0.21)	-0.32 (0.40)	-0.15 (0.32)	-0.08 (0.23)	0.16 (0.22)	-0.17 (-0.32)
**Mother's Educational Attainment (Reference—Less than high school)**							
High school	-0.20 (0.32)	0.31 (0.31)	0.35 (0.81)	0.20 (0.51)	-0.04 (0.42)	0.31 (0.32)	0.93 (0.35)
College &above	1.02** (0.36)	1.47*** (0.36)	1.99* (0.90)	1.68** (0.54)	1.12** (0.42)	1.12** (0.39)	1.33* (1.99)
**Father's Educational Attainment (Reference—Less than high school)**							
High school	0.34 (0.33)	0.60 (0.32)	0.80 (0.80)	0.88 (0.51)	0.60 (0.35)	0.66* (0.32)	0.01 (0.80)
College &above	0.90* (0.38)	1.62*** (0.38)	0.88 (0.93)	1.67** (0.54)	1.66*** (0.44)	2.07*** (0.44)	1.59* (0.88)
***Time spent on other activities***							
**Attend Extracurricular Activities? (Reference—No)**							
Yes	0.99*** (0.23)	1.20*** (0.22)	1.40** (0.44)	0.92* (0.37)	1.35*** (0.28)	1.30*** (0.28)	1.57*** (1.40)
**Average Time Spent on Homework Per Day (Reference—< 1 hour)**							
1–2 hours	-0.90* (0.39)	-0.96** (0.37)	-1.46 (0.86)	-0.88 (0.49)	-1.04* (0.43)	-1.02* (0.39)	-0.66 (-1.46)
2–3 hours	-2.15*** (0.39)	-2.43*** (0.38)	-3.62*** (0.88)	-2.90*** (0.52)	-2.42*** (0.44)	-2.31*** (0.42)	-1.57** (-3.62)
> 3 hours	-3.16*** (0.47)	-3.60*** (0.45)	-4.48*** (1.14)	-4.32*** (0.67)	-4.06*** (0.66)	-3.02*** (0.64)	-2.74*** (-4.48)
***Health Status***							
**Weight Status (Reference—Not obese)**							
Obese	-0.67* (0.32)	-0.49 (0.30)	-1.08 (0.69)	-0.18 (0.55)	-0.22 (0.41)	-0.46 (0.31)	-0.63 (-1.08)
**Having Chronic Allergic Rhinitis? (Reference—No)**							
Yes	0.32 (0.29)	0.54 (0.28)	0.45 (0.56)	0.38 (0.43)	0.09 (0.39)	0.80* (0.36)	0.84* (0.45)
**Having Asthma? (Reference—No)**							
Yes	-0.25 (0.53)	0.01 (0.50)	-1.23 (1.47)	-0.74 (1.14)	0.46 (0.60)	0.70 (0.65)	0.66 (-1.23)
**Intercept**	17.01*** (1.60)	14.08*** (1.56)	10.89 (5.90)	11.76** (4.39)	13.40** (4.23)	12.97** (4.44)	12.77* (10.89)
**R** ^**2**^	0.14	0.24					
**0.10 Pseudo R** ^**2**^			0.16				
**0.25 Pseudo R** ^**2**^				0.11			
**0.50 Pseudo R** ^**2**^					0.15		
**0.75 Pseudo R** ^**2**^						0.13	
**0.90 Pseudo R** ^**2**^							0.13

1. Bootstrapped standard errors with 100 replications.

* P<0.05

**P<0.01

***P<0.001

**Table 3:** Regression Results of the Relation between Frequency of Moderate-Intensity Physical Activity (MoIPA, days/week) and Distribution of Academic Performance Scores (N = 1470, Year = 2009).

	OLS Regression		Quantile Regression with School Effects^1^				
	Without School Effects	With School Effects	10^th^ quant.	25^th^ quant.	Median	75^th^ quant.	90^th^ quant.
	*β*(SE)	*β*(SE)	*β*(SE)	*β*(SE)	*β*(SE)	*β*(SE)	*β*(SE)
**MoIPA**	0.02 (0.04)	0.06 (0.04)	0.14 (0.08)	0.15* (0.07)	0.03 (0.06)	0.03 (0.06)	-0.06 (0.07)
**Age**	0.07 (0.15)	0.06 (0.14)	-0.07 (0.48)	0.05 (0.36)	0.17 (0.38)	0.46 (0.41)	0.60 (0.47)
***Demographic and Socioeconomic Characteristics***							
**Gender (Reference—Male)**							
Female	0.73*** (0.21)	0.76***(0.19)	0.75 (0.40)	0.48 (0.31)	0.50 (0.27)	0.71**(0.21)	1.11**(0.33)
**Household Income Per Capita Per Month (Reference—>¥2500)**							
¥<1500	0.22 (0.31)	-0.54 (0.30)	-0.43 (0.63)	-0.32 (0.51)	-0.85 (0.41)	-0.80* (0.35)	-1.15 (0.63)
¥1500–2500	0.42 (0.27)	0.01 (0.26)	0.37 (0.50)	-0.04 (0.35)	-0.02 (0.32)	-0.09 (0.32)	-0.06 (0.45)
**Family Structure (Reference—Nuclear family)**							
Single-parent	-0.38 (0.48)	-0.56 (0.45)	-0.32 (0.68)	-0.89 (0.55)	-0.73 (0.57)	-0.43 (0.85)	0.17 (0.90)
Extended family	0.05 (0.22)	-0.03 (0.21)	-0.06 (0.42)	-0.24 (0.32)	-0.07 (0.28)	0.24 (0.23)	0.03 (0.32)
**Mother's Educational Attainment (Reference—Less than high school)**							
High school	-0.26 (0.32)	0.31 (0.31)	0.01 (0.68)	0.26 (0.40)	0.15 (0.40)	0.30 (0.34)	0.87 (0.64)
College & above	0.90* (0.37)	1.46***(0.36)	1.70* (0.79)	1.62***(0.45)	1.42** (0.46)	1.01** (0.38)	1.26* (0.63)
**Father's Educational Attainment (Reference—Less than high school)**							
High school	0.30 (0.33)	0.60 (0.32)	0.94 (0.65)	0.76 (0.43)	0.55 (0.37)	0.62 (0.34)	0.06 (0.57)
College & above	0.73 (0.38)	1.61***(0.38)	0.98 (0.79)	1.44** (0.49)	1.66** (0.53)	2.14***(0.47)	1.85** (0.71)
***Time spent on other activities***							
**Attend Extracurricular Activities? (Reference—No)**							
Yes	0.95*** (0.23)	1.20***(0.22)	1.25* (0.50)	0.83* (0.38)	1.24***(0.27)	1.27***(0.30)	1.54*** (0.38)
**Average Time Spent on Homework Per Day (Reference—< 1 hour)**							
1–2 hours	-0.92* (0.39)	-0.96**(0.37)	-1.26 (0.93)	-1.12* (0.46)	-0.88 (0.46)	-1.08**(0.38)	-0.64(0.48)
2–3 hours	-2.09***(0.40)	-2.42***(0.38)	-3.35***(0.94)	-2.90***(0.55)	-2.27***(0.47)	-2.22***(0.41)	-1.62**(0.47)
> 3 hours	-3.04***(0.47)	-3.57***(0.45)	-4.25***(1.16)	-4.26***(0.51)	-3.82***(0.55)	-3.06***(0.48)	-2.54***(0.71)
***Health Status***							
**Weight Status (Reference—Not obese)**							
Obese	-0.67* (0.32)	-0.48 (0.30)	-0.90 (0.53)	-0.15 (0.47)	-0.40 (0.41)	-0.45 (0.35)	-0.59 (0.50)
**Having Chronic Allergic Rhinitis? (Reference—No)**							
Yes	0.18 (0.30)	0.52 (0.28)	0.55 (0.58)	0.24 (0.36)	0.06 (0.38)	0.89* (0.38)	0.84 (0.46)
**Having Asthma? (Reference—No)**							
Yes	-0.25 (0.53)	-0.01 (0.50)	-1.22 (1.00)	-0.64 (0.86)	0.54 (0.62)	0.57 (0.77)	0.67 (0.89)
**Intercept**	16.87***(1.61)	13.79 (1.55)	10.14 (5.32)	12.03** (3.91)	13.23**(4.04)	12.31**(4.38)	12.32* (5.12)
**R** ^**2**^	0.13	0.24					
**0.10 Pseudo R** ^**2**^			0.16				
**0.25 Pseudo R** ^**2**^				0.12			
**0.50 Pseudo R** ^**2**^					0.15		
**0.75 Pseudo R** ^**2**^						0.13	
**0.90 Pseudo R** ^**2**^							0.13

1. Bootstrapped standard errors with 100 replications.

* P<0.05

**P<0.01

***P<0.001

**Table 4:** Regression Results of the Relation between Frequency of Minimal-Intensity Physical Activity (MiIPA, days/week) and Distribution of Academic Performance Scores (N = 1470, Year = 2009).

	OLS Regression		Quantile Regression with School Effects ^1^				
	Without School Effects	With School Effects	10^th^ quant.	25^th^ quant.	Median	75^th^ quant.	90^th^ quant.
	*β*(SE)	*β*(SE)	*β*(SE)	*β*(SE)	*β*(SE)	*β*(SE)	*β*(SE)
**MiIPA**	0.12** (0.04)	0.13** (0.04)	0.24**(0.08)	0.17* (0.07)	0.11 (0.06)	0.08 (0.06)	0.00 (0.07)
**Age**	0.07 (0.15)	0.06 (0.14)	-0.05 (0.52)	0.19 (0.34)	0.20 (0.36)	0.56 (0.35)	0.45 (0.51)
***Demographic and Socioeconomic Characteristics***							
**Gender (Reference—Male)**							
Female	0.72** (0.21)	0.75*** (0.19)	0.73 (0.36)	0.65 (0.29)	0.49 (0.25)	0.73 (0.22)	1.05 (0.36)
**Household Income Per Capita Per Month (Reference—>¥2500)**							
¥<1500	0.17 (0.31)	-0.57 (0.30)	-0.22* (0.60)	-0.25* (0.49)	-1.00(0.37)	-0.95**(0.33)	-1.33**(0.64)
¥1500–2500	0.40 (0.27)	-0.01 (0.26)	0.27 (0.46)	0.03 (0.33)	-0.18**(0.31)	-0.24**(0.32)	-0.04* (0.48)
**Family Structure (Reference—Nuclear family)**							
Single-parent	-0.40 (0.48)	-0.59 (0.45)	-0.10 (0.73)	-1.06 (0.62)	-0.59 (0.62)	-0.44 (0.75)	0.15 (0.94)
Extended family	0.01 (0.22)	-0.05 (0.21)	-0.03 (0.33)	-0.56 (0.31)	-0.04 (0.25)	0.13 (0.24)	-0.11 (0.34)
**Mother's Educational Attainment (Reference—Less than high school)**							
High school	-0.24 (0.32)	0.32 (0.31)	0.93 (0.71)	0.22 (0.44)	0.11 (0.41)	0.08 (0.29)	0.83 (0.58)
College & above	0.90* (0.36)	1.48*** (0.36)	2.52** (0.75)	1.36** (0.48)	1.30** (0.50)	0.98** (0.36)	1.28 (0.66)
**Father's Educational Attainment (Reference—Less than high school)**							
High school	0.34 (0.33)	0.62* (0.32)	0.41 (0.69)	0.88 (0.47)	0.76* (0.36)	0.80* (0.36)	-0.05 (0.63)
College & above	0.74 (0.38)	1.60*** (0.37)	0.60 (0.76)	1.80** (0.59)	1.74*** (0.46)	2.20*** (0.46)	1.62* (0.68)
***Time spent on other activities***							
**Attend Extracurricular Activities? (Reference—No)**							
Yes	0.91*** (0.23)	1.17*** (0.22)	1.25** (0.42)	0.76* (0.36)	1.21***(0.29)	1.28***(0.29)	1.50***(0.39)
**Average Time Spent on Homework Per Day (Reference—< 1 hour)**							
1–2 hours	-0.93* (0.39)	-0.96** (0.37)	-1.37 (0.84)	-1.15* (0.52)	-0.99 (0.54)	-0.96**(0.35)	-0.63 (0.46)
2–3 hours	-2.12***(0.39)	-2.44*** (0.38)	-3.32*** (0.81)	-2.99*** (0.50)	-2.38***(0.53)	-2.22***(0.40)	-1.61** 0.49)
> 3 hours	-3.01*** (0.47)	-3.52*** (0.45)	-3.87*** (0.96)	-4.20*** (0.60)	-3.86***(0.66)	-3.09***(0.58)	-2.70***(0.66)
***Health Status***							
**Weight Status (Reference—Not obese)**							
Obese	-0.64* (0.32)	-0.47 (0.30)	-0.98 (0.63)	-0.32 (0.51)	-0.27 (0.38)	-0.51 (0.28)	-0.60 (0.37)
**Having Chronic Allergic Rhinitis? (Reference—No)**							
Yes	0.18 (0.29)	0.53 (0.28)	0.74 (0.47)	0.31 (0.34)	0.14 (0.41)	0.77* (0.34)	0.78 (0.43)
**Having Asthma? (Reference—No)**							
Yes	-0.19 (0.53)	0.04 (0.50)	-1.07(1.31)	-0.94 (0.82)	0.37 (0.74)	0.61 (0.69)	0.78 (0.93)
**Intercept**	16.28***(1.62)	13.32*** (1.56)	8.79 (5.72)	10.29** (3.86)	12.62**(3.97)	10.84**(3.70)	13.83**(5.27)
**R** ^**2**^	0.13	0.24					
**0.10 Pseudo R** ^**2**^			0.17				
**0.25 Pseudo R** ^**2**^				0.12			
**0.50 Pseudo R** ^**2**^					0.15		
**0.75 Pseudo R** ^**2**^						0.13	
**0.90 Pseudo R** ^**2**^							0.13

1. Bootstrapped standard errors with 100 replications.

* P<0.05

**P<0.01

***P<0.001

**Fig 1:**
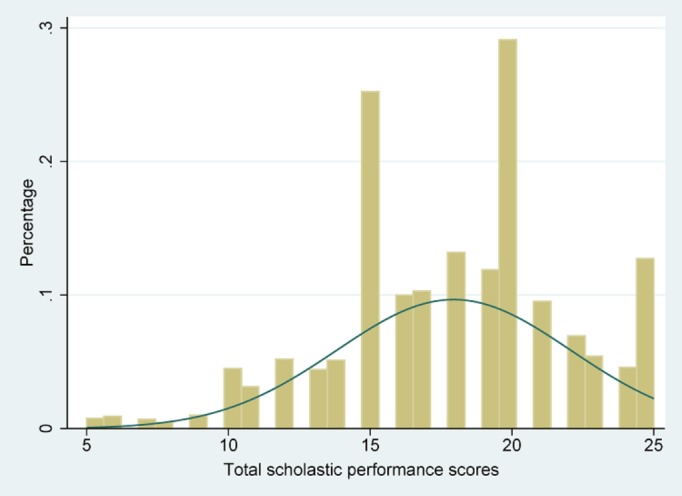
Histogram for Academic performance scores.

**Fig 2:**
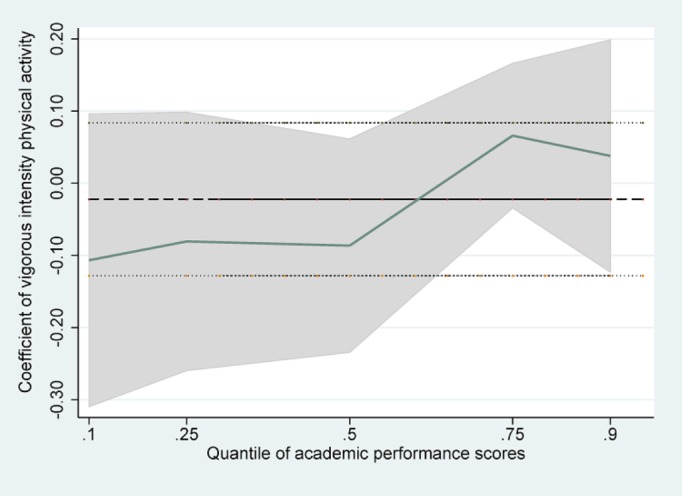
Quantile regression estimates of VIPA. Y axes represent coefficient estimates of physical activities over the distribution of Academic performance scores; X axes indicates the quantiles of the dependent variable. The dashed line is the OLS estimate for physical activities and the dotted lines are the 95% confidence interval bounds. The shaded areas represent the 95% confidence intervals for the quantile regression estimates (100 bootstrap replications).

**Fig 3:**
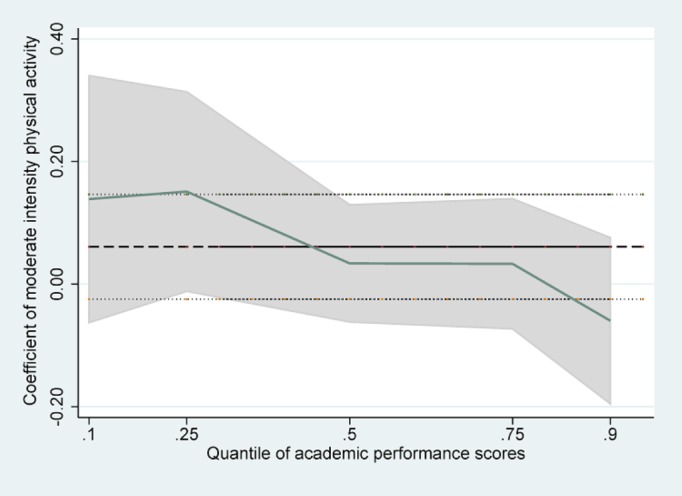
Quantile regression estimates of MoIPA.

**Fig 4:**
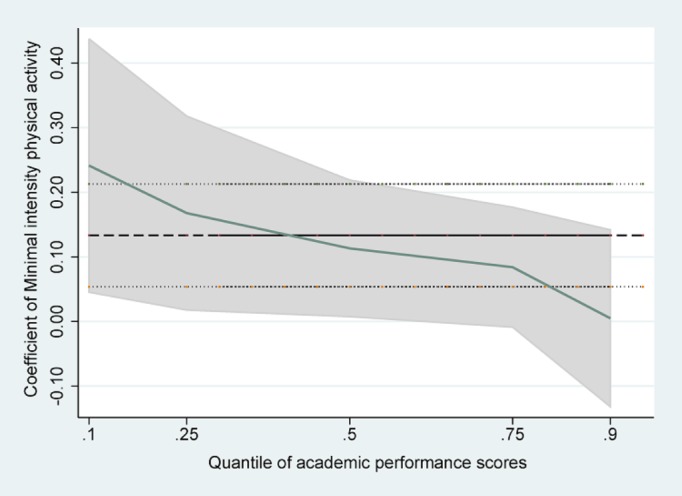
Quantile regression estimates of MiIPA.

Fig. 1 was a histogram showing the distribution of APS, from which we noticed that there were some students who belonged to the extremely lower parts of the APS distribution. Figs. 2–4 showed the coefficients of each PA variable for different quantiles of the performance scores. Focusing on Fig. 2, we can see that the association between VIPA and APS was similar throughout the full range of the APS distribution, and the quantile regression estimates was close to the linear estimate for VIPA. However, the coefficients of MiIPA appeared to be different across the spectrum of academic scores. For example, the coefficient for frequency of MiIPA at the 10^th^ percentile was larger than that at the 90^th^percentile. So the positive association between minimal physical activity such as walking and academic performance scores tended to be larger at the lower end of the distribution than at the higher end of the distribution.

## Discussion

Our study provided a more comprehensive analysis of the relationship between academic achievement and physical activity than the ordinary least squares regression. For those who belonged to the lower end of APS, the magnitude of positive association between frequency of moderate- and minimal-intensity physical activity and APS appeared to be significantly larger than for those who were at the higher end of APS. Although we could not infer causal relationship given the cross-sectional design of the study, our finding showed that physical activity could be particularly beneficial for the scholastic performance of those at the highest risk for academic failure and school dropout, even if the physical activity is only of minimal intensity.

This finding is consistent with previous studies that showed moderate physical activity can improve children’s self-esteem, physical fitness, concentration and memory[15,31,32]. One of our study’s unique strengths was its use of a representative sample of fifth-grade students in Shanghai’s public schools. We collected information on diverse social and health factors including parent’s socioeconomic status and family structure, as well as students’ health conditions and time allocation on various activities, enabling the analysis of pathways that may confound the relationship of interest. The second strength of this study was that we applied quantile regression analysis to assess the unequal association of physical activity with different ranges of academic performance scores, providing evidence for strategizing interventions for target populations. Our third unique strength is that we use teacher-reported measure to operationalize the scholastic performance, thus removing the possible biases from parent-reported or student-reported variables about academic achievement.

### Limits

The cross-sectional design of the study limits the conclusions that may be drawn. For instance, although socio-economic, family and school environmental factors were important determinants of scholastic performances; genetic endowment may have provided an inherited predisposition for some students to be successful in both athletics and academic study [33]. Also, mental health has been shown in the literature to be as an important predictor of problem behavior and poor academic outcomes. These factors were not measured and adjusted in this study. Furthermore, the self-reported measurement on different intensity of physical activity may contain information bias. Students may not recall the frequencies or over-report their frequencies of exercises in a school setting. Albeit independent of their APS, this non-differential measurement error may weaken the study findings. The teacher-reported APS may also be influenced by school policy and environment, but this bias should be attenuated when school-level fixed effects were adjusted. Finally, we found that the relationship between frequency of PA and APS varied by individual school, yet, while this study did not intend to identify specific modifiable factors at the school-level., those factors may have important implications for interventions, which should be assessed in future studies.

### Policy Implication

The findings from this study support the promotion of physical activities in schools. Those students who had poor academic performance could be worse off in their grade if schools or parents replace their physical activity time with more homework and extracurricular study time. This is particularly critical because students who did not achieve satisfactory academic scores were more likely to be assigned more homework.

Our results showed that the average time spent on homework per day, in particular more than 2 hours per day, was negatively associated with academic performance, which may reflect the real situation in Shanghai’s public schools. Thus it may make sense for schools to limit homework time within 2 hours. A guideline for studying time of primary school students released by Ministry of Health stipulates that homework for fifth and sixth graders should be within 90 minutes, which is obviously not have been fully implemented [34]. Studies in western education system shared the same insights. For example, a study based on systematic review suggested that homework be realistic in length and difficulty given the students' abilities to work independently. 5 to 10 minutes per subject might be appropriate for 4th graders [35]. Another review suggested a “10-minute” rule, according to which the appropriate homework length should be 50 minutes in fifth graders [36]. This could be particularly important for the teachers, parents and students to change their current norm of using extra homework length to improve academic achievement. Instead, moderate- and minimal-intensity physical activities may have a positive influence on academic achievement, and should be strongly encouraged.

As for the feasibility issue of how to promote physical activity among students who might have a disadvantaged background, there have been a number of guidelines recommending proven strategies to improve physical activities in schools or communities among children. U.S. Department of Health and Human Services issued a guide that recommended community-wide health education campaigns, school-based physical education, and social support in community settings, highlighting the role of multisite, multicomponent interventions in sustainably increasing physical activity behaviors [37]. The US Centers for Disease Control and Prevention promoted strategies that increase student’s physical activity in schools (e.g., a Kids Walk-to-School manual provided guidance for schools and communities on how to create an environment to support safe walking and bicycling to school) [38]. A multilevel approach to promoting physical activity is likely to be effective, which combines school-based interventions with family or community involvement, along with educational interventions with policy and environmental changes [39]. Physical activity can be increased during school break periods, through existing youth organizations, summer day camps and possibly through active transportation [40].

## References

[1] Bonell C, Humphrey N, Fletcher A, Moore L, Anderson R, et al (2014) Why schools should promote students' health and wellbeing. BMJ 13: 348 10.1186/1475-2875-13-348 25134103

[2] Trudeau F, Shephard RJ (2008) Physical education, school physical activity, school sports and academic performance. International Journal of Behavioral nutrition and Physical Activity 5(1): 10. 18298849 10.1186/1479-5868-5-10 PMC2329661

[3] Coe DP, Pivarnik JM, Womack CJ, Reeves MJ, Malina RM (2006) Effect of physical education and activity levels on academic achievement in children. Medicine and Science in Sports and Exercise 38(8): 1515 16888468 10.1249/01.mss.0000227537.13175.1b

[4] Drollette ES, Shishido T, Pontifex MB, Hillman CH (2012) Maintenance of cognitive control during and after walking in preadolescent children. Medicine and Science in Sports and Exercise 44(10), 2017–2024. 10.1249/MSS.0b013e318258bcd5 22525770

[5] Flöel A, Ruscheweyh R, Krüger K, Willemer C, Winter B, et al (2010) Physical activity and memory functions: are neurotrophins and cerebral gray matter volume the missing link?. Neuroimage 49(3): 2756–2763. 10.1016/j.neuroimage.2009.10.043 19853041

[6] Colcombe SJ, Kramer AF, Erickson KI, Scalf P, McAuley E, et al (2004) Cardiovascular fitness, cortical plasticity, and aging. Proc Natl Acad Sci U S A 101:3316–3321. 14978288 10.1073/pnas.0400266101 PMC373255

[7] Erickson KI, Voss MW, Prakash RS, Basak C, Szabo A, et al (2011) Exercise training increases size of hippocampus and improves memory. Proc. Proc Natl Acad Sci U S A 108:3017–3022. 10.1073/pnas.1015950108 21282661 PMC3041121

[8] Ploughman M (2008) Exercise is brain food: the effects of physical activity on cognitive function. Developmental neurorehabilitation 11(3): 236–240. 10.1080/17518420801997007 18781504

[9] Tremblay MS, Inman JW, Willms JD (2000) The relationship between physical activity, self-esteem, and academic achievement in 12-year-old children. Pediatric exercise science 12(3): 312–323.

[10] Sallis JF, McKenzie TL, Kolody B, Lewis M, Marshall S, et al (1999) Effects of health-related physical education on academic achievement: Project SPARK. Research quarterly for exercise and sport 70(2): 127–134. 10380244 10.1080/02701367.1999.10608030

[11] Rasberry CN, Lee SM, Robin L, Laris BA, Russell LA, et al(2011) The association between school-based physical activity, including physical education, and academic performance: A systematic review of the literature. Preventive Medicine 52 Suppl 1:S10–20. 10.1016/j.ypmed.2011.01.027 21291905

[12] Raver CC, Jones SM, Li-Grining C, Zhai F, Bub K, et al (2011) CSRP’s Impact on Low-Income Preschoolers’Preacademic Skills: Self-Regulation as a Mediating Mechanism. Child development 82(1): 362–378. 10.1111/j.1467-8624.2010.01561.x 21291447 PMC3682645

[13] Sektnan M, McClelland MM, Acock A, Morrison FJ (2010) Relations between early family risk, children's behavioral regulation, and academic achievement. Early Childhood Research Quarterly 25(4): 464–479. 20953343 10.1016/j.ecresq.2010.02.005 PMC2953426

[14] Law M, King G, King S, Kertoy M, Hurley P, et al (2006) Patterns of participation in recreational and leisure activities among children with complex physical disabilities.Developmental Medicine & Child Neurology 48(5): 337–342. 16608540 10.1017/S0012162206000740

[15] Chomitz VR, Slining MM, McGowan RJ, Mitchell SE, Dawson GF, et al (2009) Is there a relationship between physical fitness and academic achievement? Positive results from public school children in the northeastern United States.Journal of School Health 79(1): 30–37. 10.1111/j.1746-1561.2008.00371.x 19149783

[16] Perry JC, Liu X, Pabian Y (2010) School engagement as a mediator of academic performance among urban youth: The role of career preparation, parental career support, and teacher support. The Counseling Psychologist 38(2): 269–295.

[17] Griew P, Page A, Thomas S, Hillsdon M, Cooper AR (2010) The school effect on children's school time physical activity: The PEACH Project. Preventive medicine 51(3): 282–286. 20600261 10.1016/j.ypmed.2010.06.009

[18] Ministry of Education of PRC (2007) Announcement on result of the investigation of physical fitness and health of Chinese students in 2005.Health Medicine Research and Practice 4(1): 5–7.

[19] Ministry of Education of PRC (2010) Outline of Chinese medium and long term educational reform and development plan (2010–2020). Available: http://www.moe.edu.cn/publicfiles/business/htmlfiles/moe/moe_838/201008/93704.html. Accessed 29 July 2010.

[20] Ji CY, Cheng TO (2009) Epidemic increase in overweight and obesity in Chinese children from 1985 to 2005.International journal of cardiology 132(1): 1–10. 10.1016/j.ijcard.2008.07.003 18835050

[21] Ministry of Education of PRC (2011) Regulation in ensuring one-hour physical activity in school for primary and middle school students every day.

[22] Tan C (2000) Theory on aesthetics of education. Educational Research 12: 30–33.

[23] Xu Y (2002) Consideration of physical education and health education. Journal of Physical Education 9(5): 116–117.

[24] Lai AX, Liu H, Liu S (2007) Causes and restraining measures for the constant deterioration of the constitution of teenagers in China. Journal of Physical Education 14(5): 125–128.

[25] Craig CL, Marshall AL, Sjöström M, Bauman AE, Booth ML, et al(2003) International physical activity questionnaire: 12-country reliability and validity. Medicine & Science in Sports & Exercise 195(03): 3508–1381. 10.1249/01.MSS.0000078924.61453.FB 12900694

[26] Bruni O, Ferini-Strambi L, Russo PM, Antignani M, Innocenzi M, et al (2006) Sleep disturbances and teacher ratings of school achievement and temperament in children. Sleep Med 7(1): 43–8. 16309959 10.1016/j.sleep.2005.09.003

[27] Jiang YR, Chen WJ, Sun WQ, LF, Li SH, et al (2011) School achievements in school-aged children with different sleep duration and quality. Chinese Mental Health Journal 25(6): 444–448.

[28] Shanghai Bureau of Statistics. Shanghai Statistical Yearbook 2010. Available: http://www.stats-sh.gov.cn/data/toTjnj.xhtml?y=2010e.

[29] Group of China Obesity Task Force (2004) Body mass index reference norm for screening overweight and obesity in Chinese children and adolescents. Zhonghua Liu Xing Bing XueZaZhi (Chinese journal of epidemiology) 25(2): 97–102. 15132858

[30] Koenker Roger (2005) Quantileregression Cambridge university press. No. 38.

[31] Janssen I, LeBlanc AG (2010) Review Systematic review of the health benefits of physical activity and fitness in school-aged children and youth. International Journal of Behavioral Nutrition and Physical Activity 7(40): 1–16. 10.1186/1479-5868-7-40 20459784 PMC2885312

[32] Strong WB, Malina RM, Blimkie CJ, Daniels SR, Dishman RK, et al (2005) Evidence based physical activity for school-age youth. The Journal of pediatrics 146(6): 732–737. 15973308 10.1016/j.jpeds.2005.01.055

[33] Poropat AE (2009) A meta-analysis of the five-factor model of personality and academic performance. Psychological bulletin 135(2): 322 10.1037/a0014996 19254083

[34] Ministry of Health of PRC. Standards of Daily Study Time for Primary School Students. School Health 1998. Available: http://www.moh.gov.cn/ewebeditor/uploadfile/2013/03/20130313091400151.pdf.

[35] Good TL, Brophy JE (2003) Looking in classrooms (9th ed.). Boston: Allyn & Bacon.

[36] Cooper H (2007) The battle over homework (3rd ed.). Thousand Oaks, CA: Corwin Press.

[37] Kahn EB, Ramsey LT, Brownson RC, Heath GW, Howze EH, et al (2002) The effectiveness of interventions to increase physical activity a systematic review. Am J Prev Med 22(4S): 73–107. 11985936 10.1016/s0749-3797(02)00434-8

[38] Centers For Disease Control (2013) Make a Difference at Your School. Chronic Disease. 31p. Available at: http://digitalcommons.hsc.unt.edu/disease/31.

[39] van Sluijs EM, McMinn AM, Griffin SJ (2007) Effectiveness of interventions to promote physical activity in children and adolescents: systematic review of controlled trials. British Medical Journal 335: 703–707. 17884863 10.1136/bmj.39320.843947.BE PMC2001088

[40] Jago R, Baranowski T (2004) Non-curricular approaches for increasing physical activity in youth: a review. Preventive Medicine 39: 157–163. 15207997 10.1016/j.ypmed.2004.01.014

